# Incidence and Risk Assessment of Acute Kidney Injury (AKI) in Spine Surgery: A Case Report and Literature Review

**DOI:** 10.3390/jcm14041210

**Published:** 2025-02-12

**Authors:** Calogero Velluto, Giovan Giuseppe Mazzella, Laura Scaramuzzo, Maria Ilaria Borruto, Michele Inverso, Lorenzo Fulli, Matteo Costanzi, Marco Rossi, Luca Proietti

**Affiliations:** 1 Department of Aging, Orthopaedic and Rheumatological Sciences, Fondazione Policlinico Universitario Agostino Gemelli IRCCS, 00136 Rome, Italy; calogerovelluto@gmail.com (C.V.); ggmazzella@gmail.com (G.G.M.); maria.ilaria.borruto@gmail.com (M.I.B.); inversomichele7@gmail.com (M.I.); lorenzofulli@gmail.com (L.F.); luca.proietti@policlinicogemelli.it (L.P.); 2 Department of Anesthesiology and Intensive Care Medicine, Fondazione Policlinico Universitario Agostino Gemelli IRCCS, 00136 Rome, Italy; matteo.costanzi@policlinicogemelli.it (M.C.); marco.rossi@policlinicogemelli.it (M.R.)

**Keywords:** acute kidney injury, adult spine deformity, risk factors, complications, nephrotoxicity

## Abstract

**Background:** Acute kidney injury (AKI) is a critical medical condition characterized by a sudden decline in renal function, often resulting in severe complications and increased mortality. In the context of spine surgery, particularly for adult spine deformities, the risk of AKI is significant due to the complexity and duration of these procedures, as well as the substantial intraoperative blood loss and hemodynamic instability they can entail. Despite advancements in surgical and perioperative care, AKI remains a major concern. This paper presents a case report of AKI following spine deformity surgery and conducts a comprehensive literature review to evaluate the incidence and risk factors associated with AKI in this specific surgical population. **Methods**: A systematic literature search was conducted across the PubMed, Medline, and Cochrane Library databases, focusing on studies published between January 2000 and December 2023. The inclusion criteria targeted studies reporting on adult patients undergoing spine surgery, specifically detailing the incidence and risk factors of AKI. Exclusion criteria included studies on pediatric patients, non-English publications, and those lacking clear AKI diagnostic criteria. Data from the selected studies were independently extracted by two reviewers and analyzed using descriptive statistics and meta-analysis techniques where applicable. The case report highlights a patient who developed AKI following extensive spine surgery for Adult Spine Deformity (ASD), detailing the clinical course, diagnostic approach, and management strategies employed. **Results**: The literature review revealed that the incidence of AKI in spine surgery varies widely and is influenced by factors such as patient demographics, type of surgery, and perioperative management. Identified risk factors include significant blood loss, prolonged operative time, intraoperative hypotension, and the use of nephrotoxic drugs. The findings underscore the importance of vigilant perioperative monitoring and proactive management strategies to mitigate the risk of AKI. These strategies include optimizing hemodynamic stability, minimizing blood loss, and careful management of nephrotoxic medications. **Conclusions**: By integrating a detailed case report with a thorough review of the existing literature, this paper aims to enhance the understanding of AKI in spine surgery and inform clinical practices to improve patient outcomes.

## 1. Introduction

Acute kidney injury (AKI) is a significant medical condition characterized by a rapid decline in renal function that can lead to severe complications and increased mortality if not promptly recognized and managed [1]. The occurrence of AKI in surgical patients is particularly concerning due to the complex interplay of factors such as perioperative hemodynamic instability, blood loss, and the use of nephrotoxic agents [2]. In the context of spine surgery, the risk of AKI may be further exacerbated by the intricate nature of the procedures, prolonged operative times, and the potential for substantial intraoperative blood loss [3].

Spine surgery, particularly for the correction of adult spine deformities, poses unique challenges and risks. These surgeries often require extensive corrective maneuvers of the spine and this could lead to repercussions on the adjacent structures, which can contribute to significant physiological stress on the patient [4]. AKI was defined using the KDIGO (Kidney Disease: Improving Global Outcomes) consensus classification [5], which offers a structured approach to identifying and categorizing the severity of AKI. According to this guideline, AKI diagnosis is based on changes in serum creatinine levels. The classification breaks down into three distinct stages. Stage 1 AKI is identified when serum creatinine levels are 1.5 to 1.9 times the baseline value or show an absolute increase of at least 0.3 mg/dL. Stage 2 AKI is characterized by serum creatinine levels rising to 2.0 to 2.9 times the baseline. The most severe category, Stage 3 AKI, occurs when serum creatinine levels are at least three times the baseline, increase by 4.0 mg/dL or more, or when renal replacement therapy becomes necessary.

Despite advancements in surgical techniques and perioperative care, complications such as AKI remain a critical concern. Understanding the incidence and risk factors associated with AKI in spine surgery is essential for improving patient outcomes and developing effective preventive strategies [6]. While several studies have reported on the incidence of AKI in various surgical contexts, there is limited literature specifically addressing its occurrence following spine surgery for adult deformities. This paper aims to fill this gap by presenting a case of AKI following spine surgery for an adult spine deformity and conducting a comprehensive literature review of the existing literature. The objectives are to clarify the incidence and risk factors of AKI in this specific surgical population, assess current diagnostic and treatment strategies, and provide insights into improving perioperative management to mitigate the risk of AKI. By integrating a detailed case report with a thorough literature review, this paper aims to contribute to the understanding of AKI in spine surgery and highlight the importance of vigilant perioperative monitoring and management. The findings are expected to inform clinical practice and guide future research efforts aimed at reducing the incidence and impact of AKI in patients undergoing spine surgery.

## 2. Material and Methods

### 2.1. Search Strategy

**Figure 1:**
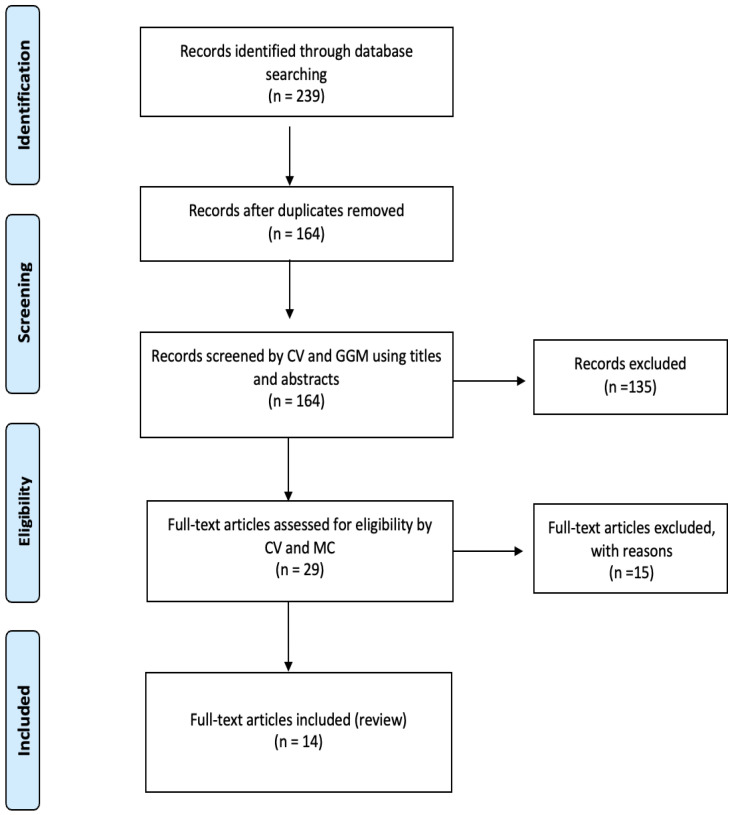
PRISMA flow-chart.

A comprehensive literature search was conducted to identify studies reporting the incidence and risk factors of acute kidney injury (AKI) following spine surgery. The databases searched included PubMed, Medline, and Cochrane Library. The search was performed using a combination of keywords such as “acute kidney injury”, “spine surgery”, “incidence”, “risk factors”, and “postoperative complications”. The search was limited to articles published in English from January 2000 to December 2023. Both prospective and retrospective studies were considered to ensure a thorough review of the available evidence. This research was conducted according to the Preferred Reporting Items for Systematic Reviews and Meta-Analyses (PRISMA) guidelines (Figure 1) [7].

### 2.2. Inclusion and Exclusion Criteria

To ensure the relevance and quality of the included studies, specific inclusion and exclusion criteria were established. The quality of included studies was assessed using the Newcastle–Ottawa Scale, focusing on the selection, comparability, and outcome domains. Bias analysis was conducted for the included studies to evaluate potential confounding factors. Studies were included if they met the following criteria:

- Reported on adult patients (age ≥ 18 years) undergoing spine surgery;
- Included data on the incidence and/or risk factors of AKI following spine surgery;
- Provided a clear definition of AKI based on established clinical criteria (e.g., KDIGO, RIFLE, AKIN).

Studies were excluded if they met any of the following criteria:

- Involved pediatric patients (age < 18 years);
- Were non-English publications;
- Did not provide clear diagnostic criteria for AKI.;
- Focused on spine surgery for trauma or oncological indications, as these may have different risk profiles.

### 2.3. Data Collection

Data extraction was performed independently by two reviewers (CV and GGM) to minimize bias and errors. The following variables were collected from each study:

- Study design (e.g., prospective, retrospective);
- Sample size and patient demographics (e.g., age, gender);
- Type and extent of spine surgery performed;
- Incidence of AKI;
- Identified risk factors for AKI (e.g., intraoperative blood loss, hypotension, use of nephrotoxic drugs);
- Diagnostic criteria used for AKI;
- Treatment strategies and patient outcomes.

Discrepancies between reviewers were resolved through discussion or by consulting a third reviewer.

### 2.4. Statistical Analysis

Descriptive statistics were used to summarize the data extracted from the included studies. The incidence of AKI was reported as a percentage with corresponding 95% confidence intervals. Data were tabulated using Numbers software Version 14.3 (Apple Inc., Cupertino, CA, USA). Categorical variables are presented as frequencies and percentages, while continuous variables are expressed as means with standard deviations. Heterogeneity among studies was evaluated using the I² statistic.

## 3. Case Report

**Figure 2:**
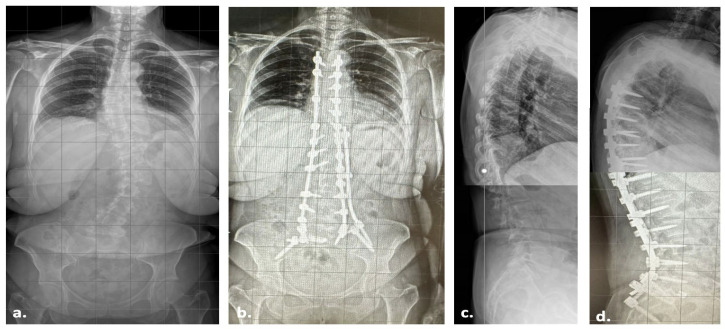
Pre- and post-operative coronal and sagittal standing position X-rays of the 61-year old female patient reported. Preoperative coronal (**a**), postoperative coronal (**b**), preoperative sagittal (**c**), and postoperative sagittal (**d**).

**Figure 3:**
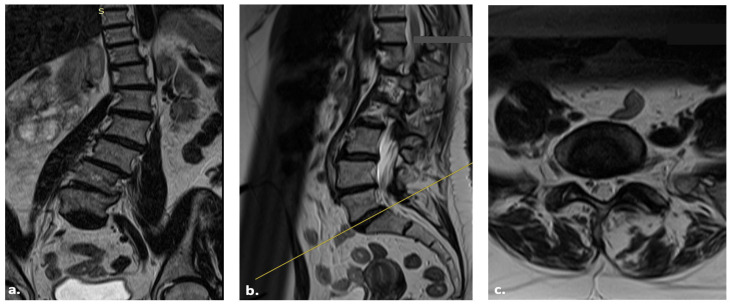
Coronal (**a**), sagittal (**b**) and axial (**c**) MRI scans of the patient showing the coronal and sagittal imbalance.

A 61-year-old female patient came to our Outpatient Clinic for Adult Spine Deformity (ASD) in March 2023. After complete preoperative evaluations, including standing position X-rays (Figure 2), CT, DEXA, and MRI (Figure 3), the patient decided to undergo posterior fusion and correction of the deformity. According to her past medical history (PMH), she had allergies to Sulfamethoxazole/Trimethoprim (Bactrim^®^, manufactured by Sun Pharmaceutical Industries Ltd., Mumbai, India) and ibuprofen. The patient had hypertension, which was under control with nebivolol, and bronchial asthma, which was being treated with formoterol/beclometasone. According to her history of present illness (HPI), she came for a visit due to dorsolumbar pain for four years, difficulty in autonomous ambulation, a left dorsal hump, and pain upon palpation in the dorsolumbar region. According to her past surgical history (PSH), she had not undergone any previous surgeries. After a multidisciplinary approach, as requested by our department, we planned a T5 to Ileum posterior fusion surgery in April 2023, with correction of the Coronal Malalignment (CM) using a Kickstand rod [8].

Surgery was performed in the prone position under general anesthesia. Electrodes for neuro-monitoring were placed by the electrophysiology staff. Baseline potentials were acquired. General anesthesia was administered for endotracheal intubation. The patient was positioned prone on a carbon fiber table with specific supports for the spine. The skin was disinfected, and a sterile field was prepared. An incision was made along the spine from T5 to the ileum using fluoroscopic guidance. The layers were dissected, and the neural arches from T5 to the ileum were exposed bilaterally. The articular masses were prepared bilaterally from T5 to the ileum to promote zygapophyseal arthrodesis. Using the Reline Open Nuvasive system and Navigation (Nuvasive, San Diego, CA, USA), polyaxial pedicle screws were placed bilaterally from T5 to S1, excluding the right side of L5 and L2. Evoked potentials remained normal throughout the procedure. Schwab 1 osteotomies [9] were performed at the curve’s apex. Fusion was completed by placing two rods, appropriately contoured to the spine’s curves that were fixed after performing corrective maneuvers. An additional iliac screw was placed and connected to an auxiliary rod on the left (according to the Kickstand rod technique) that was attached to the main left rod with two rod-to-rod connectors [8]. Neurophysiological monitoring remained stable compared to the baseline after these maneuvers. Chips of autologous bone, appropriately prepared, were placed medially and laterally to the fixation devices to promote arthrodesis. Additionally, bank bone graft (morselized femoral head) was used. Vancomycin powder was applied along the entire surgical incision, both above and below the fascia. Two subfascial drains were placed.

During all surgery the blood pressure was regular, and no hypotension was reported. Afterwards, the patient was transferred to the intensive care unit with muscle relaxation and manual ventilation at FiO_2_ = 1. She underwent comprehensive multiparametric monitoring and medical therapy. In the immediate postoperative period, she developed Stage 3 AKI, characterized by a progressive increase in serum creatinine levels and a decrease in urine output, resulting in oligoanuria. During her stay, a nephrology consultation and renal Doppler ultrasound were performed, revealing patent renal arteries and veins bilaterally.

On the third postoperative day, the patient was moved back to the general ward in good clinical condition, conscious, cooperative, and breathing normally on room air. Her hemodynamics were stable without the need for vasopressors, and she was afebrile. Although her urine output was adequate, high doses of diuretics were necessary. On this day, her creatinine was 6.53 mg/dL, BUN was 61 mg/dL, and CK was 749 U/L.

By 9 April 2023, following a phase of oligoanuria, urine output began to recover under diuretic stimulation with 20 mg of intravenous Lasix administered five times daily. Despite this, her renal function worsened further, with serum creatinine rising to 6.53 mg/dL, BUN to 61 mg/dL, sodium to 131 mmol/L, and CK decreasing to 749 U/L. Urine output recorded from 11 AM that day was about 600 mL over six hours, with no signs of peripheral edema, and she remained eupneic on room air. Blood gas analysis showed pH 7.49, HCO3- 21, BE -3.8, and K 4.6.

A renal ultrasound on April 7 revealed normal-sized kidneys with normal morphology and cortical thickness, non-dilated calyces and renal pelvis, and patent renal arteries and veins bilaterally. In consultation with the nephrologist, a 24-h infusion therapy was initiated with 1000 mL of 0.9% NaCl and 1000 mL of Ringer’s Lactate. Furosemide 40 mg was administered intravenously twice daily, with close monitoring of 24-h proteinuria. The dosage of Enoxaparine was reduced by 30% due to her current renal function, and a chest X-ray was recommended to be repeated. Renal function, urine output, weight, and electrolyte profile were closely monitored. At that time, there was no urgent indication for hemodialysis.

On 10 April 2023, a repeat chest X-ray was performed, and a thoracic surgery consultation was obtained. The chest X-ray revealed a minimal left pleural effusion, for which pleural drainage was not indicated. On April 11, bicarbonate administration was discontinued, and Ringer’s Lactate infusion was increased as per nephrology advice. Enoxaparine was stopped, and Calciparin 5000 IU was administered twice daily.

On April 12, an abdominal ultrasound documented a mild dilation of the common bile duct with a hyperechoic image suspicious for microlithiasis and the presence of biliary sludge. Deursil 300 mg was added to the therapy three times daily. The dosage of Furosemide was reduced, and Zyloric was included in the treatment.

On April 13, 24-h proteinuria and sodium levels were not significant. The estimated glomerular filtration rate (eGFR) was 24.1 mL/min. Enoxaparine 2000 IU was initiated, and Calciparin was discontinued.

On 15 April 2023, the patient was discharged home with a complete recovery of renal function. Follow-up evaluations at one, three, six, and twelve months post-surgery included thorough radiological and clinical assessments, which confirmed the stability of the spinal correction with no mechanical failures and no recurrence of kidney dysfunction according to blood tests.

## 4. Literature Review

### 4.1. Demographical Data

The demographics of patients at risk for AKI in spine surgery are varied, as demonstrated across multiple studies, and they are summarized in Table 1. Han, J. et al. conducted a retrospective cohort study, focusing on adult patients, and found that older age, male gender, and pre-existing conditions such as hypertension and diabetes significantly increased the risk of AKI [10]. Hsieh, Y.Y. et al. analyzed data from a 13-year sample of patients undergoing elective lumbar degenerative disease surgery and identified older age and male gender as notable demographic risk factors. The study reported a higher incidence of AKI in patients aged 65 and above [11].

**Table 1:** Data of patients and systematic review.

Author	Year	No. Patients	Age/Gender	AKI	Comorbidities	Surgical Treatment	Outcome
Velluto et al., this study	2024	1	61 y, F	1	Hypertension, Asthma	T4 pelvis for ASD	Complete resolution
Hsieh, Y.Y. et al. [11]	2023	424,569	61.7 y, 43.8% M 56.2% F	7108	Ischemic heart disease, Congestive heart failure, Atrial fibrillation, Diabetes, Anemia, Hypertension, Dyslipidemia, COPD, Cerebrovascular disease, Peripheral vascular disease, Overweight and obesity, Drug abuse, Alcohol abuse, Rheumatic disease, CKD, Coagulopathy	Decompression alone or fusion surgery	-
Blue, R. et al. [12]	2022	910	61.6 y, 48.9% M 51.1% F	36	-	Fusion surgery (no ACDF)	Sepsis, myocardial infarction
Chiang, T.Y. et al. [13]	2022	1833	61 y, 57.4% M 42.6% F	254	Hypertension, OPLL, Diabetes mellitus, Chronic kidney disease, Congestive heart failure, coronary heart disease	Cervical spine surgery (C0-2 5.9%, C3-T1 94.1%)	-
Han, J. et al. [10]	2021	1532	61.05 y, 61.23% M 38.775 F	126	Hypertension, Diabetes mellitus, Coronary disease, Dyslipidemia, Anemia	-	-
Farag et al. [14]	2019	1080	-	25	-	-	-
Jin et al. [15]	2019	970	61.55 y, 47% M 53% F	26	Hypertension, Diabetes mellitus	-	-
Oh et al. [16]	2020	6520	61.1 y, 50.6% M 49.4 F	248	Hypertension, Diabetes mellitus, Ischemic heart disease, Cerebrovascular disease, Anemia, Cancer	Cervical spine (25.3%), Thoracic spine (5.9%), Lumbosacral spine (68.8%)	-
Makler et al. [17]	2018	1	36 y, M	1	None	miTLIF	Rhabdomyolysis with complete resolution
Minkara et al. [18]	2017	1	18 y, M	1 (secondary to intraoperative cell salvage)	None	Posterior fusion (T2-L2) for adolescent idiopathic scoliosis	Complete resolution
You et al. [19]	2017	1	14 y, F	1 (secondary to intraoperative cell salvage)	None	Navigated T3-S1posterior fusion for Group I neuromuscular scoliosis	Complete resolution
Naik et al. [6]	2014	726	61.25 y, 41% M 59% F	28	Hypertension, Diabetes mellitus, chronic kidney disease	Elective Thoracic and/or lumbar spine surgery	-
Dakwar et al. [20]	2012	5	66 y, 80% M 20% F	5	Hypertension, Hypercholesterolemia, GERD, Chronic Obstructive Pulmonary disease, Rheumatoid Arthritis	Minimally invasive lateral transpsoas spine surgery	2 hemodialysis due to rhabdomyolysis, 3 fluid resuscitations
Papadakis et al. [21]	2008	1	52 y, M	1	Hypertension	Posterior decompression and fusion of L2-L5, discectomy and posterior lumbar interfusion at L4-L5	Full recovery after hemodialysis due to rhabdomyolysis
Okubadejo et al. [22]	2007	81	50.7 y, 9.9% M, 91.9% F	5	Hypertension, Mitral valve prolapse, Dystonia, Parkinson’s, Hypothyroidism, Coronary artery disease, Myocardial infarction, Rheumatoid Arthritis	Smith-Petersen osteotomy, pedicle subtraction osteotomy, anterior and posterior spinal fusion	4 complete resolution, 1 chronic renal failure

Naik, B.I. et al. studied a cohort of patients undergoing spine surgeries and highlighted that individuals over 65 years old and those with pre-existing renal dysfunction were more prone to develop AKI [6]. Farag, E. et al. also emphasized that patients with chronic diseases, particularly those with cardiovascular and renal comorbidities, were at an increased risk [14]. Franzén S. et al. did not focus explicitly on demographics but indicated that patient factors, including body mass index (BMI) and baseline renal function, could influence postoperative cytokine levels, which are associated with AKI development [23]. Blue, R. et al. and Chiang, T.Y. et al. both reported that patients with long-standing hypertension are at a higher risk during non-emergency decompression surgeries for conditions such as cervical spondylosis [12,13].

Oh, T.K. et al. described that intraoperative factors might have an impact on patients, suggesting that older patients might benefit more from intraoperative hypothermia prevention [16]. Jin, S.J. et al. reported that demographic factors such as a higher baseline BMI were associated with an increased risk of AKI when a prone surgical position is used [15].

Furthermore, Minkara, A.A. et al. discussed specific cases where demographics like genetic predispositions (e.g., the sickle cell trait) could interact with intraoperative factors to increase AKI risk [18]. In this case, the study reported on a young scoliosis patient with the sickle cell trait who experienced AKI following intraoperative cell salvage replacement. Similarly, Makler, V. et al. and Dakwar, E. et al. documented that individual patient characteristics, such as muscle mass and genetic factors, contributed to AKI risk, especially in the context of rhabdomyolysis [17,20].

Hsieh, Y.Y. et al. analyzed differences in AKI incidence among ethnic groups; although their specific findings were less conclusive, the study suggested that socioeconomic factors could also indirectly influence the prevalence of comorbidities, thereby affecting AKI risk [11]. Meanwhile, Okubadejo, G.O. et al. noted that the complexity of adult spinal deformity surgery might disproportionately impact older adults with multiple comorbidities, making demographic considerations essential in preoperative risk assessment [22].

### 4.2. Assessment

The assessment of patients for the risk of developing AKI during spine surgery has been a focal point in the literature. Several studies have investigated various intraoperative and perioperative factors to identify early indicators of AKI risk.

Farag, E. et al. underscored the importance of hemodynamic monitoring during surgery, advocating for careful tracking of blood pressure and urine output. This study highlighted that intraoperative hypotension is a critical factor, especially in patients with underlying cardiovascular conditions, thus indicating the necessity of maintaining hemodynamic stability throughout the procedure [14]. Conversely, Blue, R. et al. argued that intraoperative hypotension did not show a direct correlation with AKI, suggesting that other perioperative factors might be more influential [12]. Franzén S. et al. introduced a novel approach by evaluating plasma cytokine levels as an assessment tool during spine surgery [23]. They analyzed patients receiving either sevoflurane or total intravenous propofol anesthesia and found that inflammatory markers, particularly interleukins, were elevated post-surgery in patients who later developed AKI. This finding suggests that intraoperative cytokine monitoring could serve as an early predictor of AKI risk.

Oh, T.K. et al. explored the role of intraoperative hypothermia in AKI development. In their retrospective observational study, they found that maintaining normothermia during general anesthesia was associated with a reduced incidence of AKI [16]. This points to the value of temperature regulation as part of the intraoperative assessment, particularly for high-risk patients.

Han. J. et al. evaluated the impact of different anesthetic techniques on AKI risk, recommending the use of anesthesia that minimizes renal stress. They emphasized monitoring renal function markers, such as serum creatinine and urine output, intraoperatively and postoperatively to detect early signs of renal impairment [10]. Hsieh, Y.Y. et al. also recommended a comprehensive preoperative assessment that includes baseline renal function, hydration status, and evaluation of comorbid conditions like hypertension and diabetes, as these significantly impact intraoperative management and AKI risk [11].

Naik, B.I. et al. highlighted the use of the RIFLE (Risk, Injury, Failure, Loss, and End-stage kidney disease) classification system [24] for assessing AKI risk. Their study utilized changes in serum creatinine and glomerular filtration rate (GFR) as key markers for identifying patients at various stages of renal impairment [6]. This standardized assessment method allows for the stratification of patients based on their risk profile.

Chiang, T.Y. et al. reported that patients with chronic hypertension in preoperative assessment and a history of arterial hypertension showed a higher propensity for AKI in non-emergency decompression surgeries [13]. Jin, S.J. et al. examined the impact of different prone positioning on renal perfusion, suggesting that the choice of surgical positioning equipment should be part of preoperative planning [15]. Their findings indicated that a prone position could compress the renal vasculature, leading to reduced renal blood flow and higher AKI risk.

Makler, V. et al. and Dakwar, E. et al. discussed the assessment of patients for potential rhabdomyolysis, particularly in cases involving minimally invasive spine surgery. They recommended preoperative muscle enzyme testing, including creatine kinase (CK) levels, as part of the evaluation process, especially in patients with known muscle disorders or a high BMI [17,20].

Okubadejo, G.O. et al. reported that assessing patients for the potential use of antifibrinolytic agents, such as aprotinin, in complex adult spinal deformity surgeries could increase the risk of AKI [22].

### 4.3. Diagnosis

The diagnosis of acute kidney injury (AKI) following spine surgery relies on both clinical and laboratory markers, with various studies emphasizing the importance of timely identification to improve patient outcomes.

Han, J. et al. used postoperative serum creatinine levels and urine output as primary diagnostic criteria for AKI [10]. They found that even a small elevation in serum creatinine shortly after surgery could be a significant indicator of renal impairment, underscoring the importance of frequent postoperative monitoring for early diagnosis. Their study also highlighted that the occurrence of oliguria was a valuable diagnostic marker, prompting further renal function evaluation.

Naik, B.I. et al. employed the RIFLE classification (Risk, Injury, Failure, Loss, and End-stage kidney disease) to systematically diagnose AKI in spine surgery patients. This classification focuses on changes in serum creatinine, glomerular filtration rate (GFR), and urine output as markers for various stages of kidney dysfunction. Their findings suggested that utilizing a standardized diagnostic framework such as RIFLE can aid in early identification and grading of AKI severity, facilitating prompt management [6].

Jin, S.J. et al. and Oh, T.K. et al. similarly used serum creatinine and blood urea nitrogen (BUN) as markers to assess renal function postoperatively. Jin, S.J. et al. particularly focused on the impact of prone positioning during surgery and its influence on renal outcomes, indicating that postoperative elevation in these markers could signal AKI due to intraoperative renal perfusion alterations [15]. Oh, T.K. et al. highlighted that intraoperative hypothermia might influence postoperative renal function, suggesting that serial measurements of creatinine and urine output post-surgery are crucial in diagnosing AKI [16].

Hsieh, Y.Y. et al. conducted a comprehensive analysis of AKI incidence, emphasizing the utility of electronic health records (EHR) for early detection [11]. They advocated for routine postoperative serum creatinine monitoring, especially within the first 48 h, as this is the critical period for detecting AKI onset. The study also pointed out that changes in eGFR could serve as supplementary diagnostic criteria to identify AKI early in patients with pre-existing renal insufficiency.

Makler, V. et al. and Dakwar, E. et al. highlighted cases where postoperative rhabdomyolysis led to AKI. They reported that it is essential to measure serum creatine kinase (CK) levels in patients exhibiting muscle injury symptoms or presenting with dark-colored urine postoperatively [17,20]. Elevated CK levels combined with rising creatinine concentrations were identified as key diagnostic markers for AKI secondary to rhabdomyolysis, particularly in cases involving extensive muscle manipulation during minimally invasive spine surgeries.

Farag, E. et al. suggested the use of intraoperative renal perfusion monitoring as a preventive diagnostic approach, stating that maintaining stable hemodynamics during surgery could help avert postoperative renal dysfunction [14]. However, they also acknowledged the difficulty in real-time diagnosis during the operation, recommending that continuous postoperative renal function assessments are crucial for early AKI detection.

Franzén, S. et al. explored the relationship between intraoperative cytokine levels and postoperative AKI development [23]. Their study found that patients with higher plasma cytokine levels during surgery were more likely to develop AKI, proposing that cytokine profiling might serve as an early diagnostic marker for patients at risk. Although not yet a standard diagnostic tool, their findings suggest a potential role for inflammatory markers in the early identification of AKI.

Blue, R. et al. and Glassman, S.D. et al. did not focus specifically on novel diagnostic markers but emphasized the importance of using intraoperative blood pressure monitoring and postoperative renal function tests to diagnose AKI [12,24]. Both studies acknowledged the need for a comprehensive diagnostic protocol, including blood pressure management and routine serum creatinine measurements, to identify AKI promptly.

Okubadejo, G.O. et al. mentioned the risks associated with the use of antifibrinolytic agents like aprotinin, which can increase the likelihood of AKI [22]. They recommended close postoperative monitoring of renal function in patients who receive these agents, advocating for serial creatinine measurements as a diagnostic measure to detect potential renal impairment early.

### 4.4. Treatment Options

The management and treatment of AKI in patients undergoing spine surgery involve a combination of preventive measures, intraoperative strategies, and postoperative interventions to mitigate the risk and address renal impairment effectively.

Farag, E. et al. discussed the use of vasopressors during prone spine surgery, suggesting that maintaining hemodynamic stability through careful use of vasopressors could reduce the risk of AKI by ensuring adequate renal perfusion [14]. They emphasized the need for balanced fluid management to avoid both fluid overload and under-resuscitation, which could lead to renal injury. Similarly, Naik, B.I. et al. recommended goal-directed fluid therapy to maintain renal perfusion during surgery. They noted that tailored fluid management, using dynamic hemodynamic parameters to guide fluid administration, could help minimize the risk of AKI [6].

Hsieh, Y.Y. et al. focused on perioperative strategies, highlighting the importance of avoiding nephrotoxic agents, such as certain antibiotics and NSAIDs, in the treatment and prevention of AKI [11]. They suggested that careful medication management, including the use of alternatives where possible, can significantly lower the incidence of AKI in spine surgery patients. In cases where nephrotoxic drugs are necessary, close monitoring of renal function is crucial for early detection of any adverse effects.

Oh, T.K. et al. studied intraoperative hypothermia management and suggested that maintaining normothermia during surgery could reduce AKI incidence [16]. They advocated for using warming devices to prevent hypothermia, which could contribute to renal impairment, indicating that temperature regulation should be a standard part of intraoperative management for patients at high risk of AKI.

Han, J. et al. compared different anesthetic techniques and concluded that certain anesthetic methods could impact AKI outcomes. They observed that total intravenous anesthesia (TIVA) might be associated with a lower risk of AKI compared to volatile anesthetics [10]. Jin, S.J. et al. explored the effects of prone positioning apparatus on renal perfusion during spine surgery. Their findings indicated that using specific positioning devices that minimize pressure on the abdomen could enhance renal perfusion and decrease the risk of postoperative AKI. They recommended careful patient positioning during surgery as a simple yet effective intervention to support renal function [15].

Okubadejo, G.O. et al. discussed the use of antifibrinolytic agents such as aprotinin to reduce intraoperative blood loss in complex adult spinal deformity surgeries [22]. However, they cautioned against the potential risk of AKI associated with these agents. They recommended that when antifibrinolytics are used, careful monitoring of renal function postoperatively is imperative. Additionally, the study emphasized the importance of considering other blood conservation strategies, such as controlled hypotension and the use of tranexamic acid, to minimize the need for blood transfusions and thereby reduce AKI risk.

Makler, V. et al. and Dakwar, E. et al. presented cases of AKI secondary to rhabdomyolysis, a condition that can occur during spine surgery [17,20]. They suggested that in patients who develop rhabdomyolysis, aggressive hydration with intravenous fluids is crucial to prevent myoglobin-induced renal damage. They also recommended alkalinization of urine using bicarbonate infusions to reduce the nephrotoxic effects of myoglobin, thus presenting a targeted treatment approach for this specific AKI etiology.

Hsieh, Y.Y. et al. also proposed the use of perioperative statins as a preventive measure for high-risk patients, citing evidence that statins may have a renal-protective effect by enhancing endothelial function and reducing oxidative stress [11]. This pharmacological approach offers a possible avenue for reducing AKI risk in select patient populations undergoing spine surgery.

Minkara, A.A. et al. reported on AKI associated with the use of intraoperative cell salvage and transfusion. They suggested that careful assessment of cell salvage indications and strict monitoring of hemolysis markers could guide appropriate transfusion practices, thereby reducing the risk of transfusion-related AKI [18].

Blue, R. et al. and Glassman, S.D. et al. highlighted that avoiding intraoperative hypotension is a key modifiable factor in reducing AKI risk. They advocated for vigilant blood pressure monitoring and prompt intervention to maintain adequate renal perfusion throughout the surgery [12,24]. Their studies indicated that early intraoperative intervention for hypotensive episodes could improve postoperative renal outcomes, emphasizing the importance of this modifiable factor in the treatment strategy.

### 4.5. Outcomes

Han, J. et al. conducted a retrospective cohort study revealing that patients who developed AKI after spine surgery had a higher incidence of prolonged hospital stays and required more intensive postoperative monitoring [10]. Their findings indicated that AKI could lead to increased postoperative morbidity, including infections and cardiovascular events, thus contributing to longer and more complicated recovery periods.

Hsieh, Y.Y. et al. explored the short-term and long-term outcomes of patients developing AKI after elective lumbar degenerative disease surgery. They reported an increased in-hospital mortality rate and a significant rise in long-term morbidity, particularly in older patients and those with pre-existing comorbidities [11]. Their analysis showed that patients with postoperative AKI had a higher likelihood of being readmitted within 30 days due to complications such as fluid overload and renal dysfunction, highlighting the long-term health impact of AKI in spine surgery patients.

Naik, B.I. et al. found that AKI was associated with a decrease in long-term renal function in a subset of patients. Their study demonstrated that those experiencing AKI were at an elevated risk for developing chronic kidney disease (CKD) over time, emphasizing the importance of early intervention and preventive strategies to reduce the risk of lasting renal impairment [6].

Furthermore, Blue, R. et al. examined intraoperative factors influencing AKI outcomes, noting that intraoperative hypotension was not directly correlated with AKI occurrence. However, they found that patients who developed AKI despite stable intraoperative hemodynamics often faced more severe postoperative complications, such as sepsis and myocardial infarction [12]. Glassman, S.D. et al. presented evidence that intraoperative hypotension is a key modifiable risk factor for major postoperative complications [24]. Their study indicated that effective blood pressure management during surgery could lead to better renal outcomes and reduced incidence of AKI-related complications. They also noted that patients with optimized intraoperative hemodynamics experienced fewer postoperative infections and faster functional recovery.

Farag, E. et al. discussed the relationship between intraoperative vasopressor infusion and renal outcomes. They found that patients who received vasopressors to maintain hemodynamic stability had a lower incidence of postoperative AKI, resulting in improved postoperative recovery [14]. Moreover, Jin, S.J. et al. investigated the effects of different prone positioning devices on renal function. They observed that patients positioned using devices that minimized abdominal pressure had better postoperative renal outcomes, including a reduced risk of AKI [15]. Makler, V. et al. and Dakwar, E. et al. explored the outcomes of AKI secondary to rhabdomyolysis. They reported that these patients often required prolonged intensive care and had an increased likelihood of requiring renal replacement therapy, such as dialysis [17,20]. Additionally, those who experienced rhabdomyolysis-related AKI faced a higher risk of long-term renal sequelae, including the development of CKD.

Minkara, A.A. et al. documented a case where intraoperative cell salvage was associated with AKI due to hemolysis [18].

Oh, T.K. et al. evaluated the role of intraoperative hypothermia in AKI outcomes. Their study found that patients who maintained normothermia during surgery had a lower risk of developing AKI, leading to improved postoperative recovery and decreased length of hospital stay [16]. In addition, Hsieh, Y.Y. et al. further analyzed the economic and resource implications of AKI. Their study showed that patients with AKI incurred higher healthcare costs due to prolonged hospital stays, increased use of intensive care services, and a higher likelihood of requiring postoperative dialysis [11]. Finally, Okubadejo, G.O. et al. focused on the use of antifibrinolytic agents during complex spine surgeries and their association with AKI outcomes. They reported that while these agents could reduce blood loss, they might also contribute to an increased risk of AKI [22].

## 5. Discussion

The occurrence of acute kidney injury (AKI) in patients undergoing spine surgery, particularly for adult deformities, is a significant and concerning complication. AKI is associated with increased morbidity, prolonged hospital stays, and higher mortality rates [1]. This study integrates a case report with a comprehensive literature review to elucidate the incidence and risk factors of AKI in this specific patient population, aiming to inform clinical practices and improve patient outcomes. Unlike other surgical populations, patients undergoing spine deformity surgery are particularly susceptible to AKI due to extensive procedural complexity, prolonged operative times, and greater perioperative physiological stress. Moreover, the quality of studies varied, with some demonstrating limited generalizability due to small sample sizes or lack of subgroup analyses. In addition, the study by Hsieh et al., while focusing on a general lumbar spine surgery population, offers valuable insights into AKI risk factors that are shared across various types of spine surgeries, including deformity correction.

Our case report highlights a patient who developed AKI following an extensive spine deformity surgery (T4-pelvis) for Adult Spine Deformity (ASD). This case exemplifies the multifaceted nature of AKI pathogenesis, which can arise from substantial intraoperative blood loss, prolonged surgical time, and perioperative hemodynamic instability. Despite advancements in surgical techniques and perioperative care, these factors remain significant contributors to AKI. The literature review reveals an incidence of AKI in spine surgery ranging from 2% to 20% [5]. This wide range reflects differences in study designs, patient demographics, surgical procedures, and perioperative management protocols. For example, Liu et al. reported an AKI incidence of 7.6% in their cohort of adult patients undergoing major spine surgery, while Wu et al. found an AKI incidence of 5.4% in a retrospective analysis of patients undergoing thoracolumbar spine surgery [6]. These variations highlight the need for standardized definitions and diagnostic criteria for AKI, such as those provided by KDIGO, RIFLE, and AKIN.

### 5.1. Demographics and Risk Factors for AKI in Spine Surgery

Adult spinal deformity is a well-documented degenerative condition associated with progressive spinal imbalance and gait disturbances. This understanding provides critical insights into the perioperative management strategies required to mitigate complications like AKI [25].

The literature indicates that demographic factors, such as age, gender, and underlying health conditions, play a substantial role in AKI risk. In summary, the demographics of patients at risk for AKI in spine surgery generally include older age (especially above 65 years), male gender, higher BMI, and pre-existing conditions like hypertension, diabetes, and chronic kidney disease, as well as genetic predispositions [11]. This varied profile underscores the need for tailored perioperative management strategies in at-risk populations to minimize the likelihood of AKI.

Several risk factors consistently emerge from the literature as significant predictors of AKI. These include substantial intraoperative blood loss, prolonged operative times, intraoperative hypotension, and the use of nephrotoxic agents [6]. For example, Kheterpal et al. identified significant blood loss and hypotension as independent predictors of AKI in major non-cardiac surgery, findings that are applicable to spine surgery [26]. This suggests that careful blood pressure control before and during surgery is crucial for patients with a history of hypertension [13]. The use of nephrotoxic drugs, such as nonsteroidal anti-inflammatory drugs (NSAIDs) and certain antibiotics, has also been linked to an increased risk of AKI [2].

### 5.2. Intraoperative and Postoperative Management Strategies

Fluid management during surgery is a critical component in preventing AKI. Maintaining adequate intravascular volume and ensuring sufficient renal perfusion are paramount. Strategies such as goal-directed fluid therapy, which tailors fluid administration based on dynamic hemodynamic parameters, have been shown to reduce the incidence of AKI [14]. Additionally, measures to minimize blood loss, such as using antifibrinolytic agents like tranexamic acid, can be effective in reducing the risk of AKI [22]. However, Okubadejo et al. argued that while these agents can reduce intraoperative blood loss, they may also pose a risk of AKI, warranting a thorough preoperative evaluation of the patient’s renal function and blood management plan [22].

Controlled hypotension, while useful for reducing blood loss, must be carefully managed to avoid compromising renal perfusion [12]. This finding points to the importance of hemodynamic support as part of intraoperative care to enhance renal outcomes [14] Moreover, careful consideration of patient positioning during surgery can directly impact renal perfusion and postoperative renal function [15].

Postoperative care plays a vital role in AKI prevention. Early recognition and management of potential complications through routine monitoring of renal function are essential. Implementing enhanced recovery protocols, including multimodal pain management, early mobilization, and optimal hydration, can further reduce the risk of AKI. Additionally, measures such as avoiding nephrotoxic medications and ensuring proper hydration can help safeguard renal function during the postoperative period [3].

### 5.3. Diagnosis and Assessment of AKI

In summary, the diagnosis of AKI in the context of spine surgery involves monitoring changes in serum creatinine, GFR, BUN, and urine output, with additional considerations like CK levels for patients at risk of rhabdomyolysis [17]. Incorporating standardized classifications, such as RIFLE, into postoperative care protocols aids in the early detection and grading of AKI, which is critical for timely intervention [27]. Emerging markers, like intraoperative cytokine levels, may further enhance the diagnostic process; however, they require more research before being widely implemented [23].

### 5.4. Treatment and Outcomes

Overall, treatment options for AKI in the context of spine surgery are multifaceted. Key strategies include intraoperative hemodynamic stability through fluid management and vasopressor use, careful selection and monitoring of anesthetic techniques, maintenance of normothermia, patient positioning to optimize renal perfusion, and avoidance of nephrotoxic agents [10] This finding highlighted the importance of anesthetic choice in the treatment protocol, suggesting that TIVA may offer a renal-protective effect during spine surgeries. In patients at risk for rhabdomyolysis, aggressive hydration and urine alkalinization are essential [28]. Perioperative use of statins and antifibrinolytic agents should be considered cautiously, with ongoing postoperative renal function monitoring to address potential complications promptly.

Furthermore, recent studies indicate that adverse events following posterior lumbar fusion may not be fully captured within a 30-day postoperative window [29]. This highlights the need for extended follow-up periods to accurately assess complications, including AKI, and their long-term impact on patient outcomes.

In addition to perioperative risk factors, the duration of antimicrobial prophylaxis has been linked to postoperative outcomes. In fact, Porter et al. (2022) suggest that prolonged antimicrobial use may affect recovery, potentially influencing the incidence of complications such as AKI [30]. Moreover, considering the age factor, due to the kind of patients who undergo surgery for ASD, sarcopenia has emerged as a predictor of perioperative adverse events in complex spinal surgeries [31]. This condition underscores the importance of preoperative evaluation, particularly in older patients, to mitigate the risk of complications, including AKI. While this study focuses on adult deformity surgeries, it is important to note that the incidence of AKI is also significant in pediatric populations undergoing scoliosis surgery [32]. These findings further emphasize the need for tailored perioperative strategies across different age groups.

Finally, effective interventions to minimize blood loss and transfusion risk during spine surgery are vital, as outlined by Pennington et al. (2020) [33]. These strategies, such as the use of antifibrinolytics and cell salvage techniques, play a role in reducing the risk of postoperative AKI while also considering patient safety.

Overall, a tailored approach based on patient-specific risk factors and intraoperative conditions is vital to optimize renal outcomes in spine surgery patients.

This case highlighted the potential risks of certain intraoperative practices on renal outcomes. Despite the successful use of cell salvage in reducing the need for allogenic blood transfusion, they noted the importance of monitoring for hemolysis-related AKI to improve patient prognosis [18]. Furthermore, these results indicate that temperature regulation is a vital factor in improving renal outcomes post-surgery [16].

In conclusion, AKI remains a significant complication in spine surgery for adult deformities, necessitating vigilant perioperative management to mitigate risks. This study highlights the need for standardized diagnostic criteria and targeted preventive strategies. Future research should focus on developing and refining these strategies, with an emphasis on individualized patient care to further reduce the incidence and impact of AKI in spine surgery patients.

## 6. Conclusions

Acute kidney injury (AKI) remains a significant complication in adult spine deformity surgery, driven by factors such as substantial intraoperative blood loss, prolonged operative times, and hemodynamic instability. This study highlights the importance of vigilant perioperative care, including optimized fluid management, minimized blood loss, and careful use of nephrotoxic agents. A multidisciplinary approach involving surgeons, anesthesiologists, and nephrologists is essential for mitigating the risk of AKI. By understanding the incidence and risk factors, we can implement targeted preventive strategies, ultimately improving patient outcomes in this high-risk surgical population.

## Data Availability

Data are available upon reasonable request from the corresponding author.
